# METTL3 exacerbates insulin resistance in hepatocytes by regulating m^6^A modification of cytochrome P450 2B6

**DOI:** 10.1186/s12986-023-00762-z

**Published:** 2023-09-15

**Authors:** Yongqing Li, Dantong Zhang, Yinan Gao, Peijun Wang, Zejun Wang, Bingyang Zhang, Junjun Liu, Diwen Ye, Wanshan Ma, Sumei Lu

**Affiliations:** 1 Department of Clinical Laboratory Medicine, The First Affiliated Hospital of Shandong First Medical University & Shandong Provincial Qianfoshan Hospital, Shandong Medicine and Health Key Laboratory of Laboratory Medicine, Jinan, 250000 China; 2 Department of Clinical Laboratory Medicine, Shandong Provincial Qianfoshan Hospital, Cheeloo College of Medicine, Shandong University, Jinan, 250000 China; 3 School of Laboratory Medicine, Weifang Medical University, Weifang, 261000 China

**Keywords:** CYP2B6/Cyp2b10, Insulin resistance, m^6^A methylation modification, METTL3, Non-alcoholic fatty liver disease (NAFLD)

## Abstract

**Background:**

Insulin resistance (IR) in hepatocytes endangers human health, and frequently results in the development of non-alcoholic fatty liver disease (NAFLD). Research on m^6^A methylation of RNA molecules has gained popularity in recent years; however, the molecular mechanisms regulating the processes of m^6^A modification and IR are not known. The cytochrome P450 (CYP450) enzyme system, which is mainly found in the liver, is associated with the pathogenesis of NAFLD. However, few studies have been conducted on CYP450 related m^6^A methylation. Here, we investigated the role of the methyltransferase METTL3 in exacerbating IR in hepatocytes, mainly focusing on the regulation of m^6^A modifications in *CYP2B6*.

**Methods and results:**

Analysis using dot blot and epitranscriptomic chips revealed that the m^6^A modification pattern of the transcriptome in high-fat diet (HFD)-induced fatty liver and free fatty acid (FFA)-induced fatty hepatocytes showed significant changes. CYP450 family members, especially Cyp2b10, whose homolog in humans is CYP2B6, led to a noticeable increase in m^6^A levels in HFD-induced mice livers. Application of the METTL3 methyltransferase inhibitor, STM2457, increased the level of insulin sensitivity in hepatocytes. We then analyzed the role of METTL3 in regulating m^6^A modification of *CYP2B6* in hepatocytes. METTL3 regulated the m^6^A modification of *CYP2B6*, and a positive correlation was found between the levels of CYP2B6 translation and m^6^A modifications. Furthermore, interference with METTL3 expression and exposure to STM2457 inhibited METTL3 activity, which in turn interfered with the phosphorylated insulin receptor substrate (pIRS)-glucose transporter 2 (GLUT2) insulin signaling pathway; overexpression of CYP2B6 hindered IRS phosphorylation and translocation of GLUT2 to membranes, which ultimately exacerbated IR.

**Conclusion:**

These findings offer unique insights into the role that METTL3-mediated m^6^A modifications of *CYP2B6* play in regulating insulin sensitivity in hepatocytes and provide key information for the development of strategies to induce m^6^A modifications for the clinical treatment of NAFLD.

## Introduction

Insulin resistance (IR) is a multisystem disease that can increase the risk of development of non-alcoholic fatty liver disease (NAFLD), type 2 diabetes, cardiovascular diseases, and chronic kidney diseases [1, 2]. There is a close relationship between NAFLD and IR [3, 4]. An increase in the level of hepatic de novo lipogenesis (DNL) is the main reason for an increase in serum triacylglycerol (TAG) content in liver cells of patients with NAFLD. Diacylglyceride (DAG), the immediate precursor of TAG, induces IR by stimulating the activity of protein kinase C isoforms, which inhibit the activation of phosphatidylinositol 3-kinase (PI3 kinase) and Akt/protein kinase B [5–7]. Proteins involved in the fatty acid synthesis pathway interact with those involved in the insulin signaling pathway. The molecules PI3K, Akt, and insulin receptor substrate 1 (IRS-1), and their phosphorylated counterparts play key roles in the insulin signaling pathway [8, 9]. Although many studies have been conducted on IR regulation, it remains an uncontrolled condition and has a high incidence rate.

m^6^A modification refers to the methylation of the N atom at the sixth position of the base adenosine. It is the most common post-transcriptional modification, accounting for approximately two-thirds of all RNA modifications. Previous studies have shown that m^6^A modifications are the most important internal modifications of eukaryotic mRNAs and play an important role in the pathogenesis of NAFLD. RNAs harboring m^6^A modifications are involved in the regulation of metabolism of liver triglycerides (TGs). The methylated reader protein YTH domain containing 2(YTHDC2) regulates the stability of mRNAs by recognizing the m^6^A group on mRNAs, and in this way, regulates the homeostasis of liver TGs [10]. In addition, the methyltransferase 3 (METTL3) regulates the expression of PR/SET domain 16 (PRDM16), peroxisome proliferator activated receptor gamma (PPARγ), uncoupling protein 1 (UCP1) transcripts, whose downregulation can impair the maturation of brown adipose tissues, leading to IR and high-fat diet (HFD)-induced obesity [11]. Although some studies have reported the regulatory role of m^6^A modifications in IR, this remains a largely unexplored field that awaits further exploration.

The human liver contains a variety of drug-metabolizing enzymes (DMEs) that are responsible for the metabolism of endogenous substances, including steroids and bile acids, and exogenous substances. Cytochrome P450 (CYP450) participates in oxidative biotransformation and plays an important role in metabolic activities of the liver [12, 13]. In addition, CYP450 shows high levels of expression in the liver, where it contributes to drug metabolism. Moreover, CYP450 catalyzes the monooxygenation of a variety of polyunsaturated fatty acids (PUFAs) and promotes the formation of biologically active lipid mediators [14]. CYP2B6 is a member of the CYP450 family in humans, and Cyp2b10 is its homolog in mice [15] [16]. Previous studies have shown that CYP2B6 expression is associated with NAFLD. The expression of CYP2B6 is altered in liver tissues with fatty degeneration [17], and there have been reports of a link between the expression of Cyp2b10 and the risk of NAFLD in mice [18]. However, these studies did not clearly indicate a relationship between CYP2B6 expression and NAFLD occurrence.

In the present study, we aimed to explore the molecular mechanisms by which m^6^A modifications regulate IR, focusing on the role that METTL3 plays in the regulation of insulin sensitivity in the liver via m^6^A modification of *CYP2B6*. First, the mechanism of regulation of *CYP2B6* m^6^A modification by METTL3 was investigated. Then, the effects of overexpression/silencing of METTL3 or application of the METTL3 methyltransferase inhibitor STM2457 on the insulin signaling pathway were analyzed, and CYP2B6 was overexpressed as part of a rescue strategy. This study provides theoretical guidance and directions for the clinical application of m^6^A modifications in cases of IR.

## Methods and materials

### Establishment of a mouse liver IR model

C57/BL6 mice were purchased from the Experimental Animal Center of Shandong University. Mice (15 per group) were randomly divided into a test group fed on a HFD (Catalog No. D12492, Research Diets, New Brunswick) and a control group fed on a basal diet (Catalog No. D12450B, Research Diets, New Brunswick). Body weight was measured once a week and recorded. After 16 weeks, intraperitoneal insulin tolerance test (IPITT) and intraperitoneal glucose tolerance test (IPGTT) were performed, and mice in the experimental group that had become obese and developed IR (according to their blood sugar levels) were selected. The mice were dissected and their livers were used for subsequent experiments, such as detection using a gene chip, HE staining, and immunohistochemistry. All animal experiments were conducted according to protocols approved by Ethics Committee of Shandong Provincial Qianfoshan Hospital.

### Dot blotting

The RNA was diluted into three different concentrations of 200 ng/μL, 100 ng/μL and 50 ng/μL. The RNA was heated for 5 min at 72 °C to denature the RNA and disrupt the secondary mechanism. The nucleic acids were fixed by aspirating drops of each concentration onto Hybond-N membranes and cross-linked twice (1200J, 25–50s) with UV 2400UV. Membrane 1 was incubated in methylene blue for 2–5 min and washed with water for 7–10 times to visualize Actin RNA. Membrane 2 was incubated in 10 mL of 5% milk for 60 min in a shaker at room temperature. The membrane was washed for 5 min three times. m^6^A primary antibody (Catalog No.202003, Synaptic Systems, Gottingen, Germany) was incubated overnight at 4 °C. The m^6^A primary antibody was recovered and the membrane was washed for 5 min three times. The secondary antibody was incubated for 60 min and the membrane was washed for 5 min three times. Western blotting luminescent solution was incubated for 5 min protected from light and then exposed.

### Mouse m^6^A-mRNA epitranscriptomic microarray

The HFD-fed group and control group were analyzed, with three liver samples in each group. A mouse m^6^A epitranscriptomic microarray test (8 × 60K, Arraystar) was conducted in KangChen Biotech (No. G4102A, Shanghai, China). Generally, the sample preparation and microarray hybridization were performed based on the Arraystar’s standard protocols. The total RNAs were immunoprecipitated with anti-m^6^A antibody. Agilent Feature Extraction software (version 11.0.1.1) was used to analyze acquired array images. Differentially expressed m^6^A methylated RNAs or differentially expressed RNAs between two comparison groups were identified by filtering with the fold change and statistical significance (*p* value) thresholds. Hierarchical Clustering was performed to show the distinguishable m^6^A methylation or expression pattern among samples.

### Establishment of a hepatocyte steatosis model in HepG2 cells

The human hepatic cell line HepG2 was purchased from Procell Life Science & Technology Co., Ltd (Wuhan; China). Treatment with a mixture of FFAs (palmitic acid:oleic acid (PA:OA) = 2:1) (No. SYSJ-KJ006, Kun Chuang Technology, Xian; China) at 0, 0.25, 0.5, 0.75, 1, and 1.5 mM for 24 h was used to induce steatosis in HepG2 cells. After 48 h, according to the semiquantitative oil red O staining results, the optimal concentration to induce steatosis (1.0 mM) was selected and applied in subsequent experiments.

### Semi-quantitation via oil red O staining

Observation of the HepG2 cells in which a change in fat accumulation was successfully induced under a microscope revealed obvious lipid droplet formation. These cells were fixed with tissue fixative for 10 min, after which a working solution of oil red O was added and incubated for 40 min at room temperature while avoiding light, and the sample was decolorized with a 60% isopropanol decolorizing solution for 1 min to remove background color. The cells were placed under an inverted phase-contrast microscope for observation and photography. After pictures were taken, the cells were washed twice with PBS, 100% isopropanol was added for decolorization, and the cells were mixed with the isopropanol for 10 min on a horizontal shaker. The decolorizing solution was added to a 96-well plate, with each sample was added to four subwells, and the absorbance at 510 nm was measured with a spectrophotometer.

### Glucose consumption and glucose uptake

Normal HepG2 cells and FFA-induced HepG2 cells were treated as described previously. METTL3 siRNA was transfected as normal. At the end of treatment, cells were incubated with low-glucose 10% FBS-DMEM with 10–7 mol/L insulin for 4h, whereas no insulin stimulation cells were used as negative control and empty culture medium was used as blank control. For glucose consumption, glucose concentrations in the cultured medium were determined simultaneously based on hexokinase method in Cobas8000 c701 (Roche Diagnostics, Shanghai, China). After blank, the difference of glucose levels in different groups represented the glucose consumption levels. For the glucose uptake test, we conducted as described previously [19].

### Cell transfections based on METTL3 and CYP2B6 interference or overexpression

METTL3 and CYP2B6 small interfering RNA (siRNA) was purchased from Gene Pharma (Wuhan; China). A negative control (NC) siRNA group was set up. The following siRNA sequences were used:

METTL3-Homo gene Sense: 5'-CCUGUAAGUAUGUUCACUATT-3', Antisense: 5'-UAGUGAACAUACUUGCAGGTT-3'.

CYP2B6-Homo gene Sense: 5'-GCGGAAUUGUUCCUCUUCUTT-3', Antisense: 5'-AGAAGAGGAACAAUUCCGCTT-3'.

Negative control (NC) Sense: 5'-UUCUCCGAACGUGUCACGUTT-3', Antisense: 5'-ACGUGACACGUUCGGAGAATT-3'.

At the same time, METTL3 and CYP2B6 were overexpressed by LV5 lentivirus transfection and with the pcDNA3.1 plasmid, respectively (GeneChem, Shanghai; China). For the transfection experiment, Lipofectamine 2000 reagent (No. 52887, Invitrogen) was applied to promote the transfection efficiency. In 12-well plates used to culture HepG2 cells, 100 μL of Opti-MEM was mixed with 2 μL of Lipofectamine 2000 and incubated at room temperature for 5 min, after which the Lipofectamine 2000 mixture was mixed with 100 μL of Opti-MEM and 4 μL of siRNA at room temperature. After 20 min, the Lipofectamine 2000 and siRNA mixture was added to 1 ml of RPMI 1640 culture medium per well. After 6–8 h, the culture medium was changed to normal medium. After 24 h of culturing, RNA was harvested and extracted and after 48 h of culturing, protein was harvested and extracted.

### Extraction of total protein

Total protein was extracted using radioimmunoprecipitation assay (RIPA) lysis buffer containing protease and phosphatase inhibitors according to the manufacturer’s instructions. The cell suspension with added lysate was vortexed vigorously for 40 min, and the vortexed cell suspension was placed in a low-temperature centrifuge at 12,000 rpm for 15 min. The supernatant was aspirated into a new tube for subsequent protein quantification. Quick Start Bradford 1 × Dye Reagent was used (No. 5000205, Bio-Rad, USA) for protein quantification. Before use, the Bradford reagent was filtered. Different amounts of bovine serum albumin standard (BSA) were added to 1 mL of Bradford reagent to prepare standard protein samples at 0, 2, 4, 6, 8, and 16 µg/μL. The protein sample was added to 1 mL of the Bradford reagent and mixed well. The standard curve was independently drawn, and the protein samples to be tested were moved into 96-well plates for measurement of the absorbance at 590 nm. The concentration of the protein in each sample was calculated according to the value of the standard curve.

### Extraction of membrane protein

A membrane protein extraction kit (Catalog No.89842, ThermoScientific) was applied to obtain the membrane protein. A total of 5 × 10^6^ cells were collected and centrifuged at 1000 × g for 5 min, and the supernatant was then removed. A total of 400 μL of permeabilization buffer was added and incubate at 4 °C for 10 min (while still mixing), after which the sample was centrifuged at 4 °C and 16,000 × g for 15 min. The supernatant containing the cytoplasmic proteins was then transferred to a new tube to which 200 μL of solubilization buffer was added. The mixture was then pipetted and incubated at 4 °C for 30 min (mixing was continued) before centrifugation at 4 °C and 16,000 × g for 15 min. The supernatant containing the membrane proteins was then transferred to a new tube. The Bradford assay was used for protein quantification.

### Western blotting

Samples containing 15-25μg of protein were resolved on SDS-PAGE gels, transferred to Hybond P PVDF membranes, and incubated with primary antibodies overnight at 4 °C. The anti-CYP2B6 antibody (ab140609, Abcam, Cambridge, MA) was diluted1:5000; antibodies against METTL3 (Catalog No. 96391, CST, Danvers, MA), METTL14 (Catalog No. 69159, CST, Danvers, MA), VIRILIZER (Catalog No. 69159, CST, Danvers, MA), FTO (Catalog No. 45980, CST, Danvers, MA), ALKBH5 (Catalog No. 80283, CST, Danvers, MA), WTAP (Catalog No. 69159, CST, Danvers, MA), ERK (Catalog No. 4377, CST, Danvers, MA), AKT (Catalog No. 4691, CST, Danvers, MA), phosphatidylinositol 3-kinase (PI3K) (Catalog No. 4292, CST, Danvers, MA), IRS1 (Catalog No. AF2587, Beyotime, China), and Glut4 (Catalog No. 2213, CST, Danvers, MA) were diluted 1:1000; and anti-β-actin was diluted 1:2500. The bands containing different target proteins were incubated with anti-rabbit or anti-mouse secondary antibodies for 1 h at room temperature. After exposure, the differences between the strips were compared by routine laboratory protocol.

### Immunohistochemistry

Liver tissues were used to make paraffin sections for immunohistochemistry experiments. The slices were placed in different concentrations alcohol for dewaxing and a 3% hydrogen peroxide solution to deactivate endogenous peroxidase to avoid overly intense staining. The slices were placed in a citrate buffer solution (0.01M) in a pressure cooker and heated at medium–high heat for antigen repair and after 2 min the slices were cooled to room temperature. Normal goat serum was used to block the slices for 1 h to prevent non-specific binding of the antibodies to tissues. Primary antibody anti-Cyp2b10 (Catalog No. sc-73546, Santa Cruz, CA, 1:200) was added and incubated overnight at 4 °C. Secondary antibody was added and incubated at room temperature for 1 h. Diaminobenzidine was then added to promote color development, hematoxylin was used to counterstain the nuclei, and 0.5% hydrochloric acid in alcohol was used for color separation. The slices were dehydrated, mounted on slides, and observed under a microscope.

### RT-PCR and real-time PCR

Total RNA was extracted by the TRIzol method, and 2μL of RNA was used to determine the RNA concentration. Two micrograms of RNA were reverse transcribed using a RevertAid First Strand cDNA Synthesis Kit (Catalog No. K1622, ThermoScientific) to synthesize cDNA using the following conditions: 25 °C for 5 min, 42 °C for 60 min, and 70 °C for 5 min, followed by a holding step at 4 °C. APowerUp SYBR Green Master Mix Kit (No. A25742, ThermoScientific) was used to perform real-time PCR. The sequence of each synthetic primer is shown in (Table 1).

**Table 1:** The synthetic sequence information of primers used for RT-PCR

Genes	Sequences
Mouse Cyp2b10	Forward: 5’-TCCTGACCAGTTCCTGGATG-3’
	Reverse: 5’-CTGGAGGATGGACGTAAAGAA-3’
Human CYP2B6	Forward: 5’-TTCACGGTACACCTGGGAC-3’
	Reverse: 5’-ATCACACCATATCCCCGGAAG-3’
Mouse Mettl3	Forward: 5’-CTGGGCACTTGGATTTAAGGAA-3’
	Reverse: 5’-TGAGAGGTGGTGTAGCAACTT-3’
Human METTL3	Forward: 5’-TTGTCTCCAACCTTCCGTAGT-3’
	Reverse: 5’-CCAGATCAGAGAGGTGGTGTAG-3’
Mouse Mettl14	Forward: 5’-CTGAGAGTGCGGATAGCATTG-3’
	Reverse: 5’-GAGCAGATGTATCATAGGAAGCC-3’
Human METTL14	Forward: 5’-GAGTGTGTTTACGAAAATGGGGT-3’
	Reverse: 5’-CCGTCTGTGCTACGCTTCA-3’
Mouse Wtap	Forward: 5’-TAGACCCAGCGATCAACTTGA-3’
	Reverse: 5’-CCTGTTTGGCTATCAGGCGTA-3’
Human WTAP	Forward: 5’-CTTCCCAAGAAGGTTCGATTGA-3’
	Reverse: 5’-TCAGACTCTCTTAGGCCAGTTAC -3’
Mouse Virilizer	Forward: 5’-ATGTCATGGAAACTGCACCTC-3’
	Reverse: 5’-GAGTGCTGAAAACCAAACCCA-3’
Human VIRILIZER	Forward: 5’-TACTTTGAGCCCATTTCTCCTGA-3’
	Reverse: 5’-GGAATACTGTCTACTGTTCGTCG -3’
Mouse Alkbh5	forward: 5’-CGCGGTCATCAACGACTACC -3’
	Reverse: 5’-ATGGGCTTGAACTGGAACTTG-3’
Human ALKBH5	Forward: 5’-CGGCGAAGGCTACACTTACG-3’
	Reverse: 5’-CCACCAGCTTTTGGATCACCA -3’
Mouse Fto	Forward: 5’-TTCATGCTGGATGACCTCAATG-3’
	Reverse: 5’-GCCAACTGACAGCGTTCTAAG-3’
Human FTO	Forward: 5’-ACTTGGCTCCCTTATCTGACC-3’
	Reverse: 5’-TGTGCAGTGTGAGAAAGGCTT -3’
Human PPARγ	Forward: 5’-TACTGTAGGTTTCAGAAATGCC -3’
	Reverse: 5’-GTCAGCGGACTCTGGATTCAG-3’

### MeRIP-qPCR

A total of 2 × 10^7^ cells were collected and RNA was extracted by the TRIzol method. A 1% agarose gel was prepared and the quality of the RNA was measured by electrophoresis. A methylated RNA immunoprecipitation (MeRIP) kit was purchased from Bersinbio Biotechnology (Catalog No. Bes5203, Guangzhou, China). Fragmentation buffer was added to the complete RNA and the mixture was heated at 94 °C for 1 min to fragment the RNA into 100-nt fragments. m^6^A-specific antibody or IgG was added to the fragmented RNA and the mixture was incubated at 4 °C for 2 h to allow the RNA fragments to bind to the antibody or IgG. Magnetic beads were simultaneously blocked for 2 h. The blocked magnetic beads were mixed with the RNA fragments bound to the antibody; more specifically, the magnetic beads bound to the RNA fragments which were bound to the anti-m^6^A antibody. Proteinase K was used to digest the magnetic beads for 45 min and the supernatant was transferred to a new tube. The RNA was extracted, subjected to RNA quantification and reverse transcription, and used for subsequent real-time PCR experiments. Sequence information of m6A specific primers used for MERIP-qPCR was shown in (Table 2).

**Table 2:** Sequence information ofm6A sites specific primers used for MERIP-qPCR

Genes	Sequences
HumanCYP2B6-66	Forward: 5’-GTCAGACCAGGACCATGGAA -3’
	Reverse: 5’-AAGGTTTCCCAAAAGGGGCA -3’
Human CYP2B6-425	Forward: 5’-TTCTTCCGGGGATATGGTGTG -3’
	Reverse: 5’-CCGAAGCTCCTCTATCAGACAC -3’

### Detection of RNA m^6^A modifications based on LC–MS/MS

RNA m^6^A modifications contents were detected by MetWare (http://www.metware.cn/) based on the AB Sciex QTRAP 6500 LC–MS/MS platform. Generally, sample preparation and nucleosides extraction were conducted based on generally protocol. After RNA was digested into nucleosides completely, the mixture was extracted with chloroform. The resulting aqueous layer was collected for analysis with LC-ESI-MS/MS. The sample extracts were analyzed using an UPLC-ESI-MS/MS system based on optimized conditions. The service was completed by METVIARE (Wuhan, China).

### STM2457 injection experiment in vivo

C57/BL6 male mice (eight per group) were randomly divided into four groups: basal CD group, HFD + 0.9% NaCl injection group, HFD + DMSO injection group, and HFD + STM2457 (Catalog No. 2499663-01-1, Selleck Chemicals, Houston, Texas) injection group. When the mean body weight of mice in HFD group was statistically different from that of mice in the control group [(mean body weight in HFD—mean body weight in CD)/mean body weight in CD > 30%], the treatment was started once a day for one or two weeks. STM2457 was administered at a dose of 50 mg/kg and weighed prior to each injection. DMSO or saline was also administered at an equal volume. Body weight was recorded every day. Injection of STM2457 was conducted daily and totally for one or two weeks.

### Determination of liver TC and TG in mice

Livers from different groups of mice were collected and homogenized fully. TC and TG levels were assayed and compared based on the protocol for assay lab kits for TC (Catalog No. E-BC-K109-M, Elabscience, Wuhan, China) and TG (Catalog No. E-BC-K261-M, Elabscience, Wuhan, China).

### Data analyses

The data are presented as the mean ± standard error of the mean (SEM). Comparisons between the means of two groups were analyzed using a *t* test. One-way analysis of variance (ANOVA) was performed when more than two groups were compared using the SPSS 16.0 software package. A *q* test was used for further pairwise comparisons. *P* values of less than 0.05 were considered to indicate statistical significance.

## Results

### High global m^6^A methylation change in NAFLD model

**Fig. 1:**
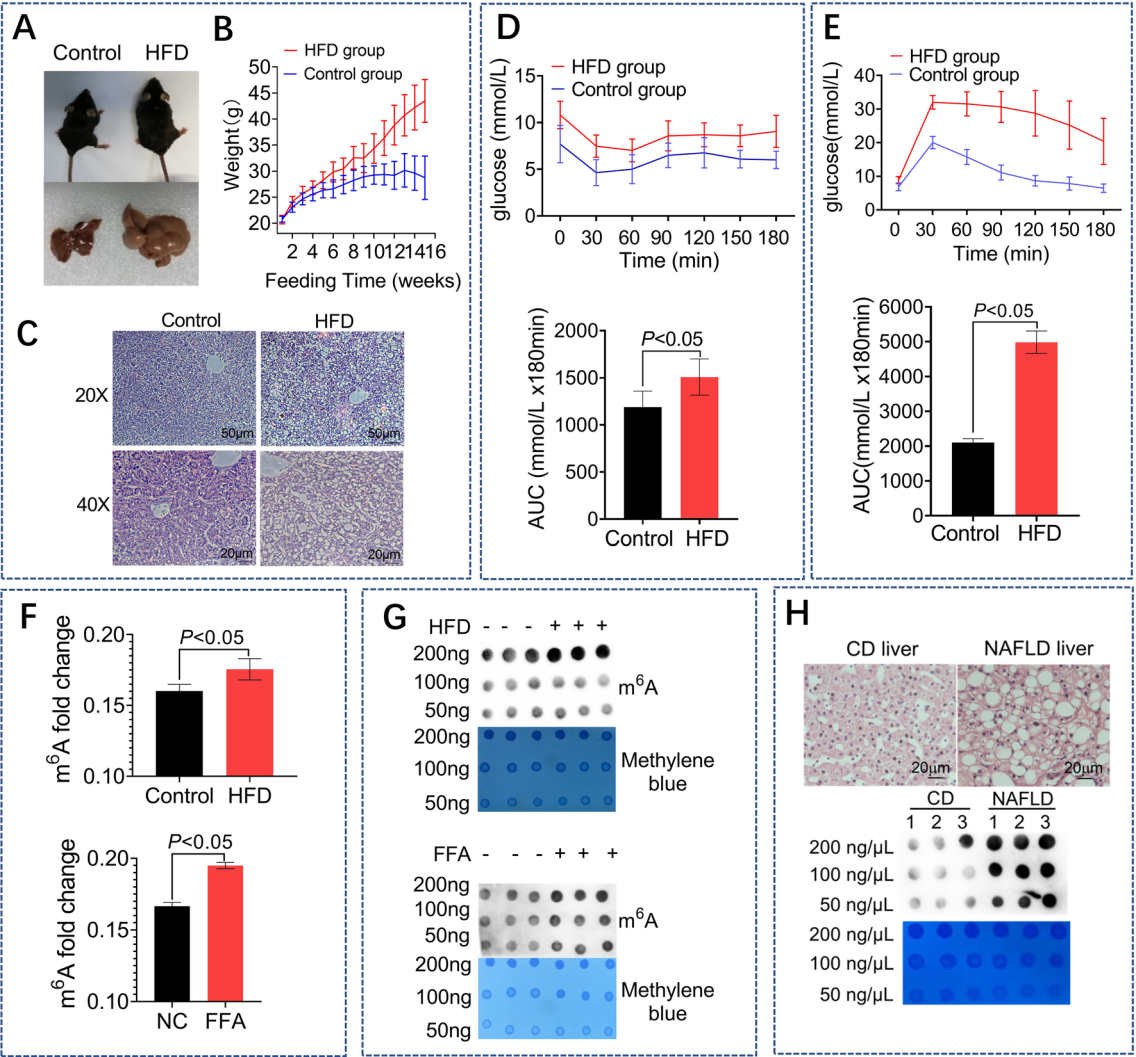
High global m^6^A methylation change in NAFLD models. A comparison of the mice body size, the mice liver size (**A**) and the mice weight (**B**) between the control group (blue) and the HFD group (red). The mice in HFD group (n = 16) were feeding with high fat diet for 16 weeks, while the mice in the control group were feeding with normal chow diet (n = 15). HE staining (**C**) (scale bars, 20 μm) of livers between the HFD and control group. The IPITT (**D**) and the IPGTT (E) tests in the HFD and control mice, the concentration of the injected insulin and glucose is 0.65 U/kg and 2.0 g/kg, respectively. LC–MS analysis (**F**) of m^6^A level in HFD mice liver and steatosis HepG2 cells (induced by 1 mmol/L FFA). Dot blot analysis (**G**) in the mice liver and in the steatosis HepG2 cells incubated with m^6^A antibodies. HE staining (scale bars, 20 μm) and the dot blot analysis (H) in the liver samples from NAFLD patients (n = 3) or CD patients (n = 3)

A NAFLD model induced by HFD based on C57/BL6 mice was induced by 16 weeks of feeding. And a steatosis HepG2 cell model was induced by free fatty acids (FFA, 1 mmol/L). Increased body size and liver volume (Fig. 1A), elevated body weight (Fig. 1B), increased growth of lipid droplets observed by HE staining (Fig. 1C), IPITT (Fig. 1D) and IPGTT (Fig. 1E) all showed obvious IR characteristics in HFD-fed mice. The Liquid Chromatograph Mass Spectrometer (LC–MS) (Fig. 1F) and Dot Blotting assay (Fig. 1G) of liver tissues in HFD-fed mice or HepG2 cells treated with FFA both showed an increase in the global levels of m^6^A methylation, indicating the potential role of m^6^A methylation in IR regulation.

Liver samples were obtained from clinical patients with and without NAFLD and analyzed by hematoxylin and eosin (HE) staining. Conspicuous lipid droplets were observed in hepatocytes obtained from the NAFLD group, whereas lipids were not observed in hepatocytes obtained from the control group. Global m6A methylation levels were elevated in fat livers (Fig. 1H).

### METTL3 increased in hepatic IR and interference of METTL3 attenuated IR induced by high fat

**Fig. 2:**
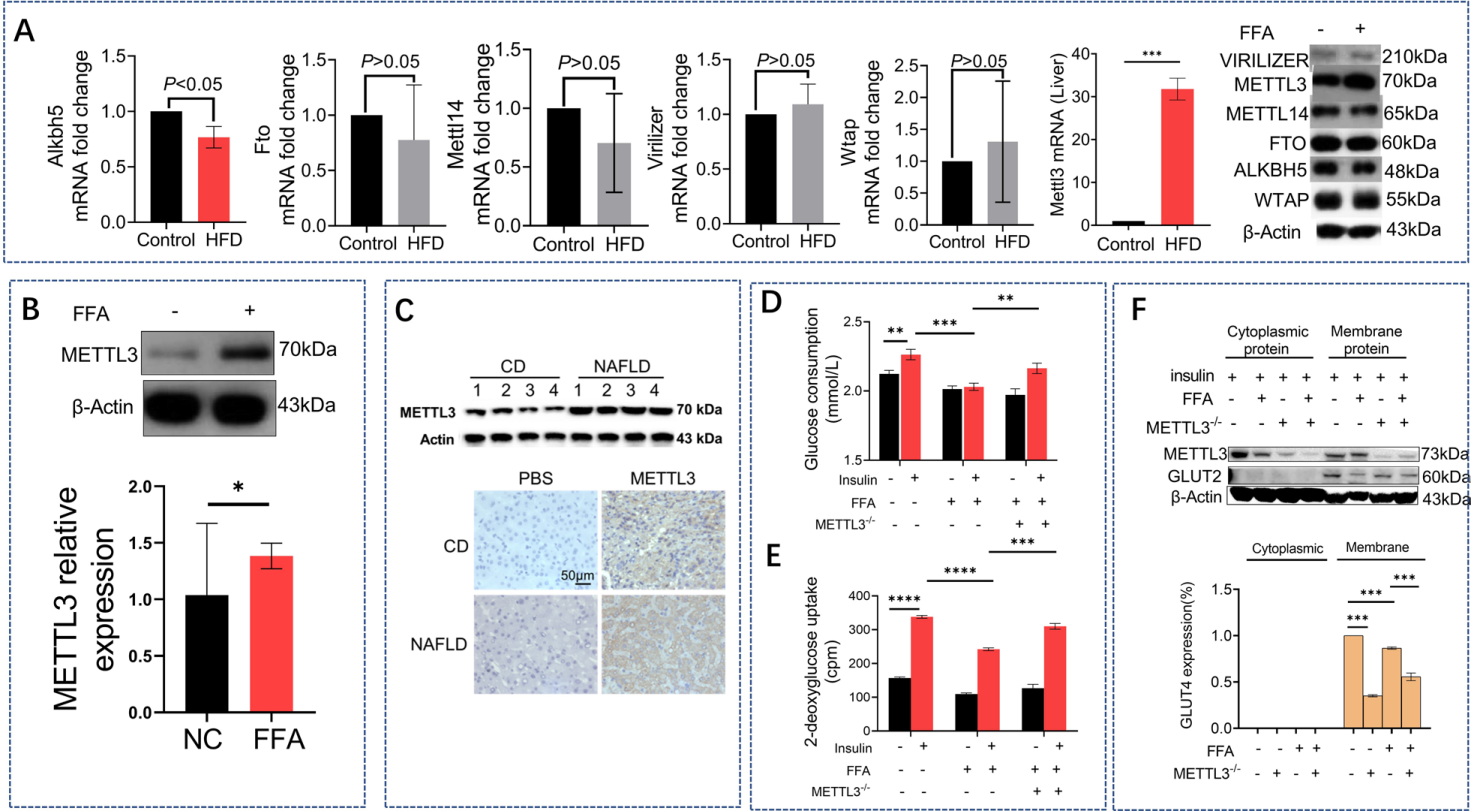
METTL3 increased in hepatic IR and interference of METTL3 attenuated IR induced by high fat. PT-qPCR analysis of all writers (Alkb5 and Fto) and erasers (Mettl14, Mettl3, Virilizer, Wtap) mRNA expression in livers from HFD mice (red) and control mice(black) (**A**). Representative western blot analysis of all writers and erasers expression in HepG2 cells between FFA group and Control group (**A**). Representative western blot analysis and quantification of Mettl3 expression in HepG2 cells between FFA group and NC group (**C**). Glucose consumption (**D**) and glucose uptake (**E**) analysis in HepG2 cells, it was divided into six groups, disposed with or without insulin; with or without FFA; with or without Mettl3 SiRNA. Representative western blot analysis and quantification of Glut2 expression in HepG2 cells disposed with insulin (10^−7^ mmol/L insulin for 4 h) between METTL3 SiRNA group and Control group (**F**)

Given the m^6^A methylation levels changes in fatty liver, we filtering all writers and erasers genes, METTL3 showed increased expression level, but not others (data not shown). PCR and Western Blot suggested METTL3 expression was upregulated in the fatty liver and in steatosis HepG2 cells (Fig. 2A, B), indicating the potential role of METTL3 in NAFLD. METTL3 levels were also examined in the livers of clinical patients by western blotting and IHC analysis (Fig. 2C). Using a previously reported method [19], we detected glucose consumption and glucose uptake in HepG2 cells. Interference of METTL3 attenuated both the decreased glucose consumption (Fig. 2D) and glucose uptake (Fig. 2E) induced by FFA treatment in HepG2 cells. Additionally, GLUT2 was shown to be mainly present in the inner cell membrane. FFA treatment significantly decreased GLUT2 levels in the membrane of HepG2 cells compared with the cytoplasmic GLUT2 protein levels. However, METTL3 interference attenuated the decrease in GLUT2 expression (Fig. 2F).

### Elevated CYP2B6 m^6^A modification and upregulation of CYP2B6 expression were identified in NAFLD model

**Fig. 3:**
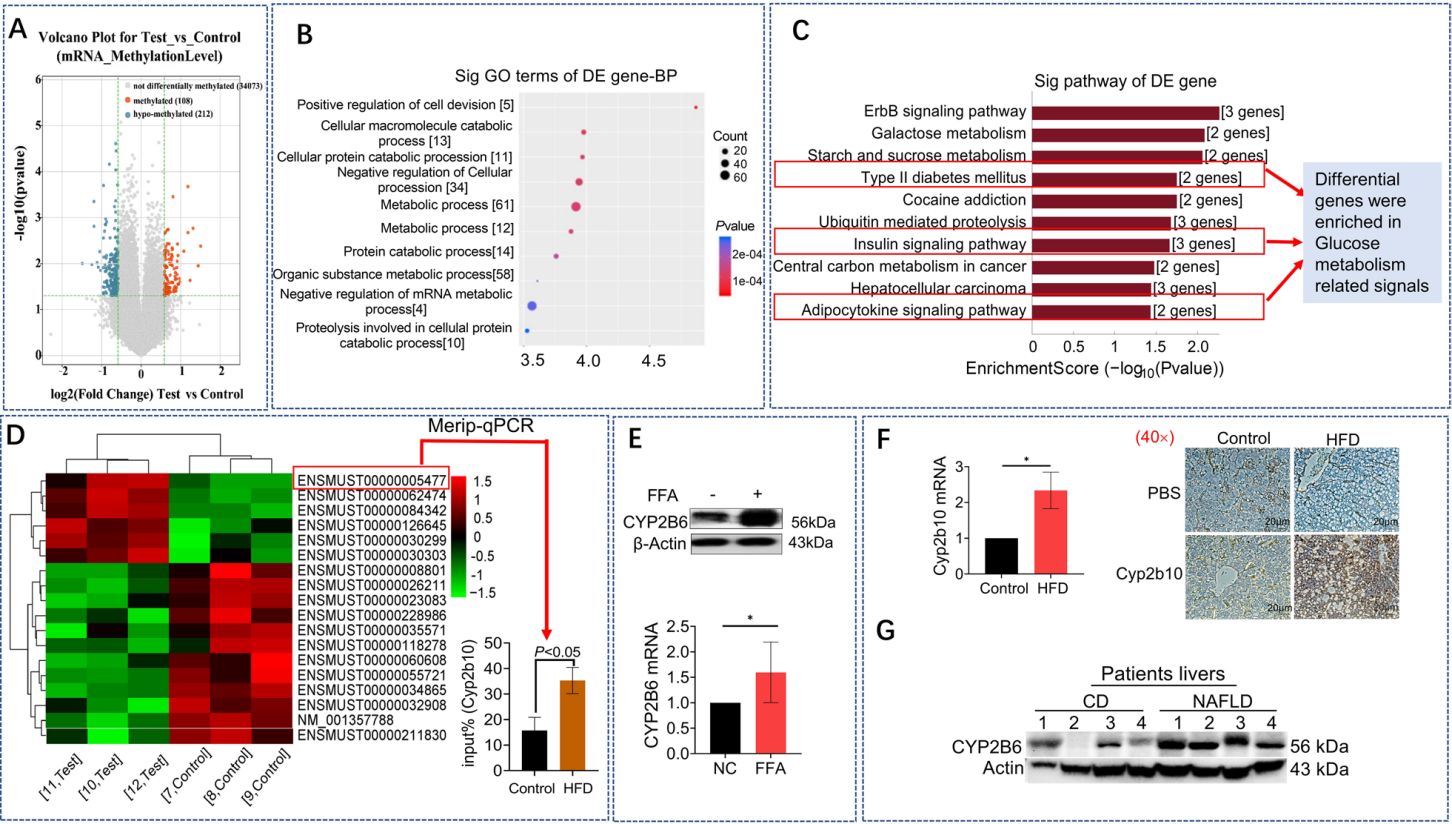
Elevated CYP2B6 m6A modification and upregulation of CYP2B6 expression were identified in NAFLD model. A mouse m^6^A epitranscriptomic microarray test of the NAFLD mice liver between HFD and control group, of all the mRNA detected, 108 hypermethylated and 212 hypomethylated genes were changed significantly in HFD-fed group (**A**). The GO and pathway analysis of the methylated mRNA showed metabolic process were enriched (**B**) and in Type II diabetes mellitus, insulin signaling pathway and adipocytokine signaling pathway (**C**). Between all the methylated mRNA, CYP450 family genes were plotted in the heatmap, the Merip-qPCR analysis showed Cyp2b10 (ENSMUST00000005477) m^6^A level was elevated (**D**) in the HFD group. IHC staining and quantitative analysis of Cyp2b10 in mice liver from HFD group and control group. Scale bars, 20 μm (**E**). A RT-qPCR analysis of Cyp2b6 between HFD group and control group and a western blot analysis of CYP2B6 (Homologous to mouse Cyp2b10 in HepG2. A western blot analysis of CYP2B6 in patients’ liver between the NAFLD group (n = 4) and the CD group (n = 4)

A mouse m^6^A epitranscriptomic microarray test was conducted. Basal diet-fed mice were used as the control group and HFD-fed mice were used as the test group, with three independent mice individuals applied to each group. The scatter plot showed m^6^A modification of differentially expressed genes occurring in the livers of mice in the HFD-fed group. For the mRNA methylation, there were 108 hypermethylated and 212 hypomethylated genes which were changed significantly in HFD-fed group (Fig. 3A).

Gene ontology analysis was done and associated the differentially expressed m^6^A methylated mRNAs enriched to certain gene ontological functions and gene ontology (GO) terms (http://www.geneontology.org). Biological process (BP), cellular component (CC) and molecular function (MF) were analyzed. Top ten hypermethylated genes related GO terms in BP are shown in Fig. 3B. Many differential genes were enriched in metabolic process and mRNA metabolic process. Pathway analysis was also performed, the differentially expressed m^6^A methylated RNAs were enriched in certain biological pathways. Figure 3C showed the top ten hypermethylated genes related pathways. The insulin signaling pathway, type II diabetes mellitus and adipokine signaling pathways were all enriched. Table 3 showed the differentially expressed genes subjected to GO and pathway analyses in Fig. 3B, C.

**Table 3:** The differentially expressed genes subjected to GO and pathway analyses in Fig. 3B, C

Gens in Fig. 3B				
Term	Gene Count	Fold enrichment	P value	GENES
Positive regulation of cell division	5	16.47	1.35067E-05	BTC//FGFR2//MEN1//TGFA//YBX1
Cellular macromolecule catabolic process	13	3.41	0.000105433	FAP//CTSF//SPOP//USP4//BAG6//UBA6//UBR3//SMARCC1//UBE2U//FMR1//YBX1//CIDEA//LAMP2
Cellular protein catabolic process	11	3.94	0.000107941	FAP//CTSF//SPOP//USP4//BAG6//UBA6//UBR3//SMARCC1//UBE2U//FMR1//LAMP2
Negative regulation of cellular process	34	1.86	0.000114543	CD36//CTBP1//FGFR2//MEN1//MYEF2//SPOP//YBX1//SPEN//CHCHD3//MTDH//FMR1//CBLB//MID1IP1//CDKN3//PALM//BTC//HK1//TGFA//TSC22D1//BAG6//ACVR1//FAP//ZBTB33//CIDEA//IFRD1//DYRK1A//USP4//SMARCC1//LAMP2//PTBP3//LINGO1//IFT80//GNAI1//ANAPC15
Metabolic process	61	1.42	0.000121324	CD36//CTBP1//FGFR2//MEN1//MYEF2//SPOP//YBX1//SPEN//CHCHD3//MTDH//TGFA//UBE2U//USP4//DYRK1A//FMR1//CWC22//RPL22//CBLB//UBA6//UBR3//HK1//SLC3A2//GANC//CYP2B10//GMPS//ERI3//CIDEA//SMARCC1//TSC22D1//NR2C2//ZFP37//ZSCAN12//ZBTB33//RPRD1B//PTAFR//PTBP3//NUP155//FYTTD1//ACVR1//KSR2//NAA50//DPM3//FAP//CTSF//BAG6//AMDHD1//MID1IP1//HACD2//LAMP2//PALM//NT5C3B//SLC2A4//CES1G//TSN//CDKN3//DCUN1D4//IFRD1//ZFP384//TMEM55B//GSTM3//ACSS3
Protein catabolic process	12	3.56	0.000133031	FAP//CTSF//SPOP//USP4//BAG6//UBA6//UBR3//SMARCC1//UBE2U//CBLB//LAMP2//FMR1
Macromolecule catabolic process	14	3.06	0.000174471	FAP//CTSF//SPOP//USP4//BAG6//UBA6//UBR3//SMARCC1//UBE2U//FMR1//CBLB//LAMP2//YBX1//CIDEA
Negative regulation of mRNA metabolic process	4	13.18	0.00024599	DYRK1A//PTBP3//YBX1//FMR1
Organic substance metabolic process	58	1.42	0.00027179	CD36//CTBP1//FGFR2//MEN1//MYEF2//SPOP//YBX1//SPEN//CHCHD3//MTDH//TGFA//UBE2U//USP4//DYRK1A//FMR1//CWC22//RPL22//CBLB//UBA6//UBR3//HK1//SLC3A2//GANC//CYP2B10//GMPS//ERI3//CIDEA//SMARCC1//TSC22D1//NR2C2//ZFP37//ZSCAN12//ZBTB33//RPRD1B//PTAFR//PTBP3//NUP155//FYTTD1//ACVR1//KSR2//NAA50//DPM3//FAP//CTSF//BAG6//AMDHD1//MID1IP1//HACD2//PALM//NT5C3B//SLC2A4//CES1G//TSN//DCUN1D4//IFRD1//ZFP384//TMEM55B//LAMP2
Proteolysis involved in cellular protein catabolic process	10	3.81	0.000296476	SPOP//USP4//BAG6//UBA6//UBR3//SMARCC1//UBE2U//FMR1//FAP//CTSF

Gens in Fig. 3C				
Definition	Fisher-Pvalue	SelectionCounts	Enrichment_Score	Genes
ErbB signaling pathway—Mus musculus (mouse)	0.005466467	3	2.262293	BTC//CBLB//TGFA
Galactose metabolism—Mus musculus (mouse)	0.008203942	2	2.085977	GANC//HK1
Starch and sucrose metabolism—Mus musculus (mouse)	0.00870976	2	2.059994	GANC//HK1
Type II diabetes mellitus—Mus musculus (mouse)	0.01787266	2	1.747811	HK1//SLC2A4
Cocaine addiction—Mus musculus (mouse)	0.01787266	2	1.747811	GNAI1//SLC18A2
Ubiquitin mediated proteolysis—Mus musculus (mouse)	0.02099069	3	1.677973	CBLB//UBA6//UBE2U
Insulin signaling pathway—Mus musculus (mouse)	0.02179601	3	1.661623	CBLB//HK1//SLC2A4
Central carbon metabolism in cancer—Mus musculus (mouse)	0.03329764	2	1.477587	FGFR2//HK1
Hepatocellular carcinoma—Mus musculus (mouse)	0.03643455	3	1.438487	GSTM3//SMARCC1//TGFA
Adipocytokine signaling pathway—Mus musculus (mouse)	0.03702735	2	1.431477	CD36//SLC2A4

Using bioinformatic tools, we scanned and identified 112 CYP family genes and created cluster heat maps for 18 genes with significant differences in m^6^A modifications levels (*P* < 0.05) (Table 4). Among these genes, *Cyp2b10* (ENSMUST00000005477) showed the greatest increase in the level of m^6^A modifications compared to that in the control group; results of methylated RNA immunoprecipitation real time fluorescence quantitative PCR (MeRIP-qPCR) analysis verified this finding (Fig. 3D). An upward trend was observed in the expression levels of *CYP2B6* (a gene homologous to *Cyp2b10* in humans) in HepG2 cells subsequent to FFA-induced fat degeneration (Fig. 3E). IHC test results confirmed overexpression of CYP2B10 in liver tissues of HFD mice (Fig. 3F). Furthermore, a similar upward trend in the expression of CYP2B6 was observed in the livers of patients with NAFLD (Fig. 3G), suggesting the potential role of CYP2B6 in hepatic metabolism.

**Table 4:** Top ten hypermethylation or hypomethylated CYP family mRNA information

Transcript_ID	Type	Regulation	Fold change	*P* value (unpaired t-test)	FDR	Gene Symbol
ENSMUST00000126645	Protein_coding	Hyper	1.3641138	0.0222037	0.4480289	Cyp4a31
ENSMUST00000005477	Protein_coding	Hyper	2.7213746	0.0112089	0.4199098	Cyp2b10
ENSMUST00000062474	Protein_coding	Hyper	1.2050972	0.0011335	0.3719519	Cyp8b1
ENSMUST00000084342	Protein_coding	Hyper	1.2079125	0.0020894	0.4127342	Cyp4a32
ENSMUST00000030299	Protein_coding	Hyper	1.330366	0.0135159	0.4224345	Cyp2j5
ENSMUST00000030303	Protein_coding	Hyper	1.3850063	0.0367759	0.4657982	Cyp2j6
NM_001357788	Protein_coding	Hypo	0.874955	0.0022313	0.4127342	Cyp4f14
ENSMUST00000055721	Protein_coding	Hypo	0.5825944	0.0085533	0.4199098	Cyp2d40
ENSMUST00000034865	Protein_coding	Hypo	0.8069894	0.0009762	0.3703641	Cyp1a1
ENSMUST00000211830	Protein_coding	Hypo	0.8845691	0.0217087	0.4456274	Cyp2c44
ENSMUST00000035571	Protein_coding	Hypo	0.879363	0.0464967	0.4750222	Cyp3a59
ENSMUST00000008801	Protein_coding	Hypo	0.878006	0.0273105	0.4551307	Cyp4f15
ENSMUST00000118278	Protein_coding	Hypo	0.8178247	0.0459065	0.47486	Cyp4a29
ENSMUST00000026211	Protein_coding	Hypo	0.8051967	0.0032982	0.4199098	Cyp2c44
ENSMUST00000023083	Protein_coding	Hypo	0.8450449	0.0232853	0.4498364	Cyp2d22
ENSMUST00000032908	Protein_coding	Hypo	0.7839067	0.0191123	0.4396346	Cyp2r1
ENSMUST00000228986	Protein_coding	Hypo	0.8361952	0.0241417	0.4500086	Cyp2d22
ENSMUST00000060608	Protein_coding	Hypo	0.7304229	0.0300979	0.4565687	Cyp20a1

### METTL3 upregulated CYP2B6 m^6^A modification and positively regulate CYP2B6 expression

**Fig. 4:**
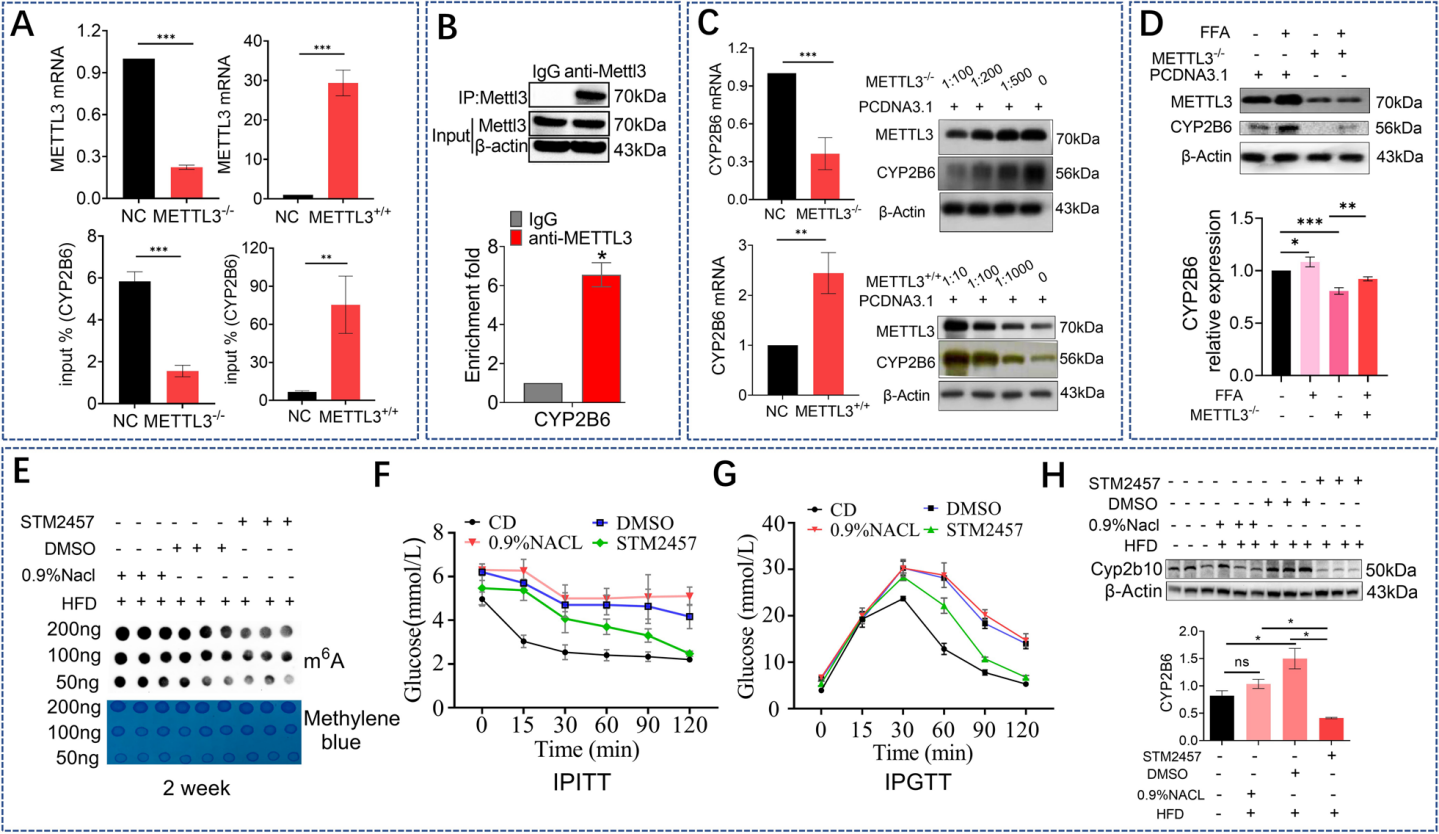
METTL3 upregulated CYP2B6 m^6^A modification and positively regulate CYP2B6 expression. Merip-qPCR analysis of the m^6^A level of CYP2B6 between the METTL3 down-expression group (infected with METTL3 SiRNA) and the METTL3 over-expression group (transfected with LV5 lentivirus) (**A**). RIP assay was performed using anti-METTL3 antibodies (**B**). RT-qPCR analysis and western blot analysis of CYP2B6 expression in HepG2 between the group of METTL3 down-expression (METTL3 SiRNA) and the group of METTL3 over-expression (METTL3 LV5 lentivirus) (**C**). Representative western blot analysis and quantification of CYP2B6 expression in HepG2 from FFA + METTL3^−/−^ group, NC + METTL3^−/−^ group and FFA group (**D**). Dot blot test in mice liver from HFD + STM2457 group, HFD group, HFD + 0.9% NaCL group and HFD + DMSO group (**E**). Both IPITT and IPGTT results showed the increased insulin sensitivity in STM2457 group (**F**/**G**). Representative western blot analysis and quantification of CYP2B6 expression in HepG2 from HFD + STM2457 group and control group (**H**)

Next, we focused on METTL3 by interfering with expression or overexpressing METTL3 in HepG2 cells. The m^6^A methylation level of CYP2B6 decreased after interference with METTL3 using siRNA (*P* < *0.001*) and increased after METTL3 overexpression (*P* < *0.01*) (Fig. 4A). RIP assay was performed using anti-METTL3 antibodies, and CYP2B6 level was observed to be increased compared with that of anti-IgG, indicating the binding of CYP2B6 to METTL3 (Fig. 4B). This suggests that METTL3 plays an important role in the m^6^A methylation of CYP2B6. After METTL3 interference, the transcript level of CYP2B6 in the METTL3^−/−^(siRNA) group decreased and METTL3 transcript levels were increased in the METTL3 overexpressing cells (Fig. 4C). Also, we showed that the CYP2B6 protein level was positively related to METTL3 expression. In fatty HepG2 cells, the increase in CYP2B6 protein expression induced by FFA treatment was attenuated by METTL3 interference (Fig. 4D).

After two weeks of treatment with STM2457 (50mg/kg, i.p.), the level of global m^6^A methylation induced by HFD in the liver showed a downward trend due to the inhibition of METTL3 activity (Fig. 4E). Both IPITT and IPGTT results showed the increased insulin sensitivity in STM2457 group, indicating that STM2457 treatment attenuated the insulin resistance status induced by HFD (Fig. 4F/G). Compared with the HFD control group, the protein expression level of Cyp2b10 in the HFD-fed group increased, however after STM2457 was administered, the protein expression level of CYP2B6 showed a decreased trend (Fig. 4H).

### Overexpression of CYP2B6 antagonize METTL3-mediated hepatic insulin resistance by regulating pIRS/IRS expression

**Fig. 5:**
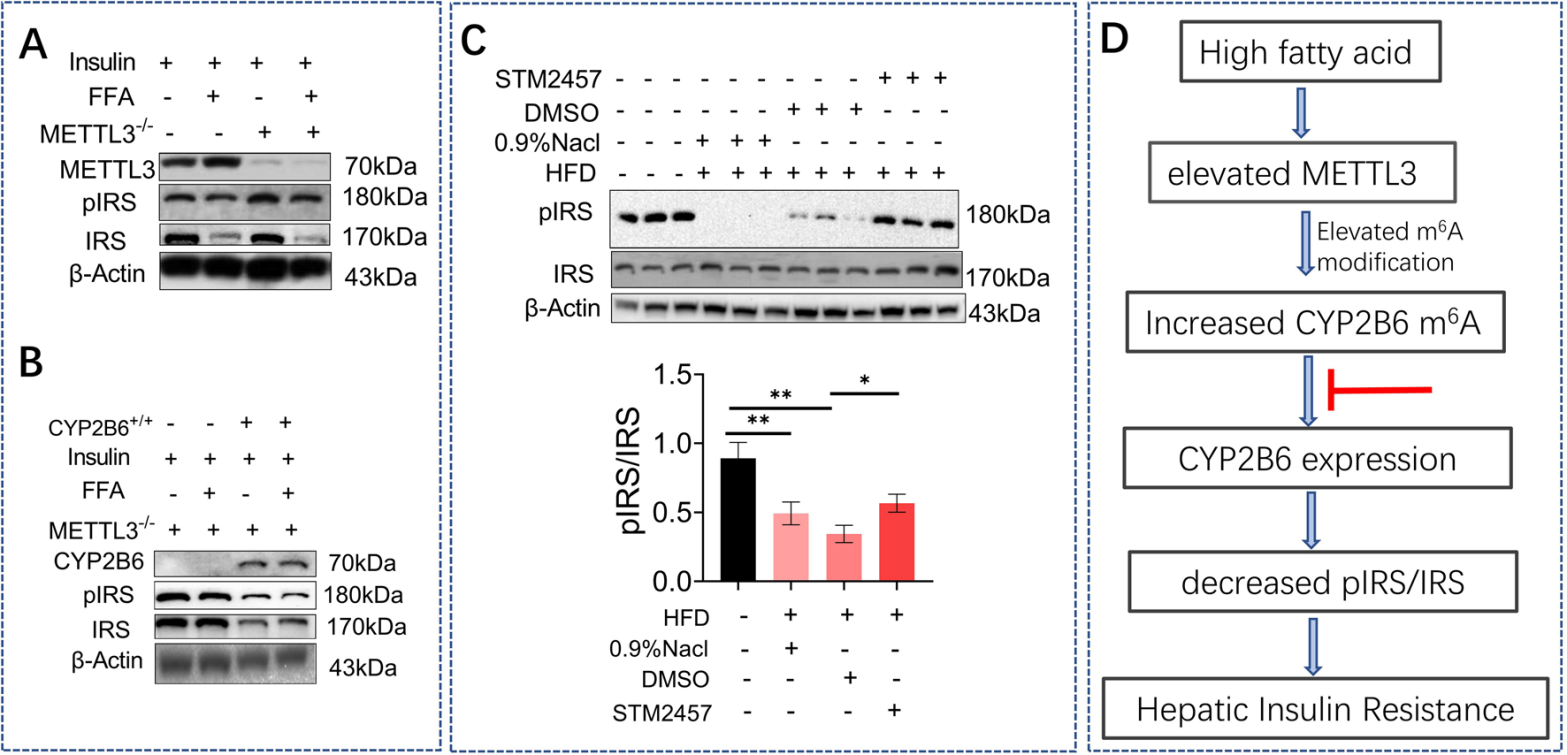
Overexpression of CYP2B6 antagonize METTL3-mediated hepatic insulin resistance by regulating pIRS/IRS expression. Representative western blot analysis and quantification of IRS and pIRS expression in HepG2 from FFA group, METTL3^−/−^group and FFA + METTL3^−/−^ group (**A**). Representative western blot analysis and quantification of IRS and pIRS expression in HepG2 from FFA group, CYP2B6^+/+^group and FFA + CYP2B6^+/+^ group (**B**). Representative western blot analysis and quantification of IRS and pIRS expression in mice liver from HFD + 0.9% NaCL group, HFD + DMSO group and HFD + STM2457 group (**C**). A schematic model related with the present study (**D**)

Phosphorylation of the insulin signaling pathway-related genes IRS were found to decrease under FFA treatment and were increased in the METTL3^−/−^ group (Fig. 5A). Overexpression of CYP2B6 antagonized the effect of METTL3 interference on the phosphorylation of IRS (Fig. 5B). These results confirmed the correlation between METTL3-mediated m^6^A methylation of CYP2B6 and insulin signaling pathway. Phosphorylation of the IRS were found to decrease under FFA treatment and were increased in the STM2457 group (Fig. 5C).

Based on the present data, we conclude a schematic model related with this study (Fig. 5D). Generally, high fatty acid induced the NAFLD phenotype in liver, with elevated METTL3 and m^6^A modification levels. Elevated METTL3 induced CYP2B6 m^6^A methylation, which further induced CYP2B6 expression. Overexpressed CYP2B6 then decreased pIRS/IRS, which is important in insulin signaling pathway, and lead to final hepatic insulin resistance. The present study indicated the potential role of CYP2B6 m^6^A methylation in hepatic IR, enriched the research of CYP2B6.

## Discussion

The present study focused on the biological function of METTL3-mediated m^6^A methylation in IR, focusing on METTL3-mediated CYP2B6 m^6^A modification. To the best of our knowledge, this is the first report focusing on CYP2B6 m^6^A modification in METTL3-mediated hepatocyte insulin sensitivity. We proved that CYP2B6 m^6^A modification is regulated by the methyltransferase METTL3. Increased expression of CYP2B6 suppressed the phosphorylation of IRS and ultimately affecting IR.

When an RNA sequence remains unchanged, chemical modification of the RNA regulates the molecular function of the RNA, thereby regulating the occurrence and development of biological activities, the process is called epitranscriptomics [20]. Among these modifications, modification with m^6^A is the most common modification of mRNAs, long noncoding RNAs (lncRNAs) and microRNAs (miRNAs) in eukaryotes [21, 22]. m^6^A methylation is involved in all aspects of RNA metabolism, including pre-mRNA splicing, 3' end processing, nuclear export, translation regulation, mRNA attenuation, and noncoding RNA processing [23–25]. These aspects have been proven to be involved in stroke [26], tumorigenesis [27], stem cell differentiation [28], regulation of the stress response [29], and other pathophysiological processes. m^6^A methylation mainly occurs on the RRACH motif, and the modification process is carried out by methylene bodies containing m^6^A methyltransferases (writers), methylation readers (readers) and demethylases (erasers) [30]. Studies have proven that m^6^A methylation plays a specific role in the pathogenesis of NAFLD. Huili Wang et al. proved that YTHDF1 and YTHDC1, m^6^A reading proteins, are abnormally expressed in the pathogenesis of NAFLD due to exposure to endocrine-disrupting chemicals [31]. Changes in the expression of METTL3 are reported to be related to steatosis, but opinions on the regulation of steatosis by METTL3 different [32]. Studies have shown that the protein expression of METTL3 is increased in HFD-fed mice [33], while other studies have shown that METTL3 is negatively correlated with the onset of NAFLD [34]. Our results showed that the expression of METTL3 was increased in a mouse model of NAFLD induced by HFD feeding, which is consistent with the FFA-induced steatosis of HepG2 cells. However, unlike the existing literature on the increased expression of FTO in NAFLD [35], our experiment showed no significant change in FTO expression. ALKBH5 also showed significant decreased here, which was not studied further in the present study.

Although m^6^A methylation has recently been a hot research topic, there are limited studies on m^6^A methylation of the CYP450 enzymes. A study from Japan on the m^6^A modification of CYP2C8 has shown that the YTHDC2 promotes CYP2C8 mRNA degradation via recognizing the m^6^A in CYP2C8 mRNA in 2019, and it leads to differences in drug metabolism between individuals [36]. Three years later, the same team showed that CYP2B6 mRNA is m^6^A modified, and METTL3 upregulated CYP2B6 expression by altering the chromatin status [37]. Our study explored the relationship between the m^6^A methylation of CYP2B6 and its relation to the pathogenesis of NAFLD for the first time. The study revealed an increase in Cyp2b10 expression in an NAFLD mouse model. In an in vitro cell model, the mRNA and protein levels of CYP2B6 were also increased. Previous studies have shown an increase in Cyp2b10 in mice with fatty liver [12] and the NAFLD inducer, perfluorooctanesulfonic acid, was shown to induce hepatocyte CYP2B6 activation in vitro [16], which is consistent with our conclusion.

We also found that the increased expression of CYP2B6 was positively correlated with the level of m^6^A modulation by METTL3 and led to IR, which has not been reported in the literature. After METTL3 interference and insulin stimulation, the expression of proteins in the insulin signaling pathway changed, and phosphorylated IRS levels inhibited by HFD increased significantly, indicating that METTL3 affects the phosphorylation of IRS. In the next experiment, we knocked down METTL3 and overexpressed CYP2B6, which reduced phosphorylation of IRS. The phosphorylation levels of insulin signaling pathway under CYP2B6 overexpression group decreased, which verified our hypothesis.

A paper reporting m^6^A methylation could affect fat metabolism showed that the administration of resveratrol to mice decreased the level of m^6^A methylation, which caused a decrease in TG levels, body weight, and fat production [38]. STM2457 is a potent inhibitor of METTL3/METTL14 catalytic activity with an IC_50_ of 16.9 nM. STM2457 is highly specific and shows no inhibition of other RNA methyltransferases. Studies have shown that the use of STM2457 can lead to reduced METTL3 activity and inhibit the growth of acute myelocytic leukemia [39]. We injected STM2457 into the HFD-fed mice and found that fat metabolism was affected. The mice lost weight, insulin resistance was relieved based on IPGTT and IPITT assay, lipid expenditure increased, TC and TG production in the liver was reduced, and the number of lipid droplets in the liver was significantly lower (data not shown). However, we did not observe an effect of m^6^A methylation on the role of lipid droplet generation in an in vitro HepG2 steatosis model induced with FFA. This may be due to the fact that the free fatty acid species used in vitro are relatively homogeneous and do not fully mimic the lipogenic effects caused by FFAs in the body.

There is little doubt that CYP2B6 may regulate IR through other mechanisms, other than insulin signaling pathway molecules, which could be indicated based on some other research. For example, as a member of the CYP450 family, CYP2E1 expression tends to increase upon HFD feeding [40]. Diabetes and oxidative stress are closely related, and a previous study showed that the overexpression of CYP2E1 in diabetic individuals could enhance NADPH oxidase activity, resulting in increased production of the reactive oxygen species, superoxide and hydrogen peroxide, by redox cycling of endogenous and exogenous substrates[41, 42]. Oxidative stress may sequentially lead to inflammation and IR [43–45]. We hypothesize that the overexpression of CYP2B6 and its high degree of m^6^A modification may also enhance NADPH oxidase activity in a similar way so that the phosphorylation levels of insulin signaling pathway genes may change and IR occurs. Of course, more evidence is under promotion, and the mechanism related data will be shown in the future work.

Some limitations existed in the present study. Experiments focusing on readers and erasers are lacking in this study, and such experiments would further clarify the CYP2B6 m^6^A modification process*. *In vivo studies based on both dysfunctional m^6^A methylation and CYP2B6 tissue-specific knockouts will undoubtedly add clarity to the biological functions in NAFLD treatment. Additionally, the role of m^6^A methylation in the catalytic activity of CYP2B6 will offer additional research insight and enhance interest in the field.

## Conclusion

In conclusion, our study proves that the methyltransferase, METTL3, can regulate m^6^A methylation of the CYP2B6 gene, antagonize insulin sensitivity through the insulin signaling pathway, and ultimately lead to the occurrence of IR in NAFLD.

## Data Availability

The data that support the findings of this study are available in the methods and supplementary material of this article.
